# Surgery

**Published:** 1859-11

**Authors:** Samuel W. Gross


### Bi-Monthly Abstracts;

OR, BI-MONTHLY NOVELTIES IN MEDICAL, PATHOLOGICAL, SURGICAL, OBSTETRICAL, PHYSIOLOGICAL, AND CHEMICAL SCIENCE.

### SURGERY.

BY SAMUEL W. GROSS, M.D.

I.—*Aneurism of the Ischiatic Artery; ligature of this vessel, and subsequently of the Primitive Iliac Artery*, with *remarks* By L. A. Dugas, M.D., Professor of Surgery in the Medical College of Georgia. (Southern Medical and Surgical Journal, October, 1859, p. 651.)

Aneurism of the ischiatic artery is an occurrence of such great rarity, and presents so much obscurity of diagnosis, as to render the case of Dr. Dugas one of great interest, especially as the ischiatic artery was ligated for its cure—an operation seldom resorted to in the living subject.

The patient, a male, twenty-four years of age, and enjoying good health, when four years old fell upon his nates; and five years subsequently perceived a small tumor near the tuberosity of the ischium, which has gradually increased and pulsated for several years. When he consulted Dr. Dugas in March, 1857, the tumor was about the size of a goose's egg, and was situated upon the inner cheek of the nates, extending in the direction of the ischiatic artery. It could be emptied by pressure; pulsated strongly; was the seat of the aneurismal bruit and thrill along the course of the ischiatic artery; and pressure upon the artery at the sciatic notch arrested both the thrill and pulsation, which returned when the pressure was removed.

The ischiatic artery was exposed by an incision, five inches in length, being made in the middle of a line extending from the tuberosity of the ischium to the posterior superior spinous process of the ilium. The ligation of the vessel put an immediate stop to the pulsations of the tumor, and the wound was united with quill sutures.

The case progressed favorably until the morning of the ninth day, when, while the patient was at stool, secondary hemorrhage suddenly came on, which was controlled by pressure. After the lapse of forty-eight hours, on account of the oozing of blood from the wound, the dressings were removed, when the blood gushed out, necessitating the opening of the wound, a procedure which showed that the blood issued a little above the ligature. The hemorrhage was arrested by plugging with lint, and the patient was much exhausted from the loss of blood. A consultation having been held, it was determined to ligate the common iliac, and Dr. W. J. Holt performed the operation. On account of the patient vomiting several times during the procedure, the peritoneum was wounded. The patient died in about fifty-eight hours, not having fairly rallied from the loss of blood. An autopsy was not made.

II.—*On Section of the Supra-Orbital Nerve in Spasms of the Eyelids.* By PROFESSOR VON GRAFE. (Archiv für Ophthalmologie, IV. 2.)

In this paper the author divides the cases of blepharospasm, in which the section of the supra-orbital nerve is indicated, into the following five classes:—

1. Cases in which persistent and excessive spasms of the orbicularis muscle are caused by the presence of a foreign body between the lids and the ball of the eye. Dr. V. Gräfe operated on two patients thus affected, with complete success.

2. Cases where a periodically returning blepharospasm developed itself in consequence of an obstinate neuralgia of the supra-orbital nerve, resisting all modes of treatment. In one instance the operation was perfectly successful, but was followed by disagreeable itching within the limits of the anæsthetic region, which, however, disappeared at the expiration of eight weeks.

3. Blepharospasms which were primarily symptomatic of a keratitis, but which persisted after the affection of the cornea was cured. Twelve persons, eight being children, were operated upon, and, with one exception, the cures were satisfactory. In those cases where it was possible to learn in which of the two eyes the spasms had originated, the nerve of the affected side was alone divided; and in most instances the muscular contractions ceased first on the operated side, and always in the upper lid sooner than the lower. If it was impossible to discover exactly where the spasm had commenced, the operation was performed on the side where the inflammation of the cornea was most severe. In a case of this description, the section of the nerve did not remedy the spasms of the orbicular muscle of the opposite side, so that the operation had to be repeated.

Dr. V. Gräfe acknowledges that cases in which it is necessary to have recourse to this means are extremely rare; the twelve in which he was obliged to operate occurring in eight thousand cases of ophthalmia, which were almost invariably accompanied by blepharospasm. He recommends for the treatment of these cases, among other remedies, immersions of the face in cold water, which cure patients in whom the spasm has existed for several months.

4. Cases where the spasm of the lids accompanies keratitis, and becomes so violent that it interferes with the treatment, and might consequently occasion loss of the eye. In three children thus affected, the operation arrested the symptom in one, and relieved it very considerably in the other two.

5. Cases in which the blepharospasm is an element of an inveterate affection of the supra-orbital nerve. The author obtained but temporary success in six patients suffering from this complication.

III.—*New Treatment of Hydrocele.* By JAMES YOUNG, M.D., Surgeon, Edinburgh. (Medical Times and Gazette, July 30th, 1859.)

The author reports several cases of hydrocele cured by the iron-wire seton, and seems to have great confidence in this mode of treatment, which was first suggested by Professor Simpson. His reasons for considering it an improvement upon the silk seton, and injections of irritating solutions, are, that it produces less pain, and effects a more speedy cure. Our own experience, which accords with that of Dr. Gillespie, as reported in a recent number of the above periodical, is, that metallic setons are either unsuccessful, or prolong the treatment, and that they compare very unfavorably with the ordinary seton.

IV.—*On Bloody Tumors of the Pavilion of the Ear, in the Insane.* By M. ACHILLE FOVILLE. (Gazette Hebdomadaire, Nos. 29 and 30, pp. 450 and 469, 1859.)

The pathology of this affection, which occurs exclusively in the insane, and especially in those affected with paralysis, is surrounded with obscurity, notwithstanding the researches of MM. Foville, Ferrus, Motet and others, who have recently published accounts of their researches in the above journal. The paper of M. Foville is very elaborate, and we have only space to extract his summary, which embodies all the practical points known on the subject:—

1. Bloody tumors of the pavilion of the ear, observed in the insane, are formed by the effusion of blood beneath the perichondrium, which is detached from the cartilage.

2. The detached perichondrium retracts upon itself in proportion as the effused blood is absorbed, and drawing with it other portions of the pavilion, thus accounts for the deformity consequent upon this class of tumors.

3. The perichondrium deposits, on its internal surface, a cartilage of new formation, which produces at one time a layer, united to its whole extent, at other times independent depots more or less separated from each other. These new products are the cause of the thickening of the ears, which have been the seat of these effusions.

4. The formation of sanguineous tumors of the ear is very frequently preceded and accompanied by general disturbance of the cephalic circulation; and it is worthy of remark that the increase of discoloration, heat, and pain, which is observed in these affections, resembles in a striking manner those symptoms seen in animals in which the sympathetic nerve has been divided in the neck, or from which the superior cervical ganglion has been removed. Although it is impossible to draw any definite conclusions from this coincidence, yet new researches in this direction may throw some light upon the etiology of congestions and hemorrhages of different parts of the head.

V.—*Report on Amputation of the Penis for Epithelial Cancer.* (Medical Times and Gazette, October 1st, 1859, p. 334.)

One of the most interesting features of the valuable periodical in which we find this report is, that from time to time it presents a collection of cases from hospital practice, on a surgical or medical topic, from which important deductions are drawn. In the report on epithelial cancer of the penis are presented short histories of thirty-five cases of this affection, operated upon in a period of more than four years. The value of these statistical reports would be much enhanced were it possible to trace the result of the cases after their discharge from the hospital. In a few instances only can this be accomplished, and hence it cannot be stated how long the patient survives, or whether the disease returns in any other portion of the body. The comments upon these cases are extracted in full:—

*Fatality of the operation.*—Amputation of the penis is not, under ordinary circumstances, an operation attended by any material risk to life. Of the thirty-five cases, all excepting two resulted in recovery; and in one of the latter the fatal event was in no way connected with the operation, and did not occur until three months after it. In the only one in which death *bona fide* resulted from the operation, it was caused by a low form of pneumonia which supervened in the fourth week. The patient had previously been a healthy man. Such results will, of course, occasionally follow all operations, even the most trivial, especially when performed in the wards of the hospital, where erysipelas, pyæmia, etc. are often rife. The risk attending amputation of the penis is probably increased slightly in proportion as the advanced stage of the disease requires that it be performed low down. When, as we have seen in one or two instances, it is required to dissect away the crura of the organ from their attachment to the bone, the danger attending it may even become considerable. In none of the cases of our present series, however, was so extensive a dissection required, and a majority of them were amputations not much below the glans.

*Age most liable to cancer of the penis.*—The age of the patient is stated in only twenty-three out of our cases. The oldest in the series was 77, the youngest 27. To classify them according to age, we find one under 30; four between 30 and 40; eight between 40 and 50; four between 50 and 60; five between 60 and 70; and one between 70 and 80. Thus it would appear that middle age is the period of life most liable to this form of cancer, a circumstance to which, probably, several influences contribute.

*Exciting causes of cancer of the penis.*—It has long been a matter of observation among surgeons, that congenital phymosis often acts as a localizing or exciting cause of cancer of the glans. The disease is exceedingly rare in Jews. In only four of the cases of our series do the notes state that this condition of the prepuce had existed; but it must be borne in mind that no special attention was given to the reporting of the previous history of the case by those who have supplied to us the data we have used. The object alone in view was the result of the operation. Thus it is highly probable that a far larger proportion were really the subjects of phymosis. In one case it is stated that a long-standing venereal sore had preceded the malignant one, and in another, the cancerous ulceration attacked a cicatrice left by a wound in infancy.

VI.—*On Buccal Cancer in Smokers.* By M. BOUISSON. (Montpellier Médical, June and July, 1859; Gazette Hebdomadaire, 1859, No. 32, p. 506.)

The learned professor of Montpellier has recently, in an elaborate article, evinced much zeal and talent in defending the old and should-be exploded idea, that cancer and cancroid affections of the lip are produced by the irritant action of tobacco incessantly concentrated upon the same point of the mucous membrane. In support of his belief he adduces sixty-eight cases of cancroid or cancer of the lips, occurring in patients who smoked to excess. He states that these affections have become much more common since the habit of smoking has become so general, and that, before the increased consumption of tobacco, they were but little known on this portion of the body. Although M. Bouisson grants that cancer of the lip may arise from other causes than smoking, yet he maintains that if a person be predisposed to this affection the use of tobacco will provoke its appearance, when it might have never shown itself under other circumstances. The short clay pipe and bad tobacco used by the poor, will develop the disease sooner than the fine cigar smoked by those who can afford it. Those who believe in this doctrine should take heed of this point.

VII.—*On the Treatment of Spina Bifida by Injections of Iodine.* By DANIEL BRAINARD, M.D., Professor of Surgery in the Rush Medical College. (Chicago Medical Journal, September, 1859, p. 517.)

In this essay Dr. Brainard, after discussing the seat and pathology of hydrorachitis, considers the different modes of treatment instituted for its relief, and gives the results of fifty-nine cases treated by puncture, excision, ligature, and pressure. From his table, we find that of 35 cases in which punctures had been made, 17 resulted in cures, 16 in death, 1 was palliated, and the result of 1 was unknown: 11 cases were subjected to excision, with 7 cures, 3 deaths, the result of 1 being unknown. Ligation was instituted in 8 instances, 5 being cured, and 3 terminating fatally. In 5 cases, pressure gave the result of 3 cures, 1 death, and 1 palliation.

The treatment of spina bifida by injections of iodine was first instituted by the author, and he illustrates his paper by ten cases treated in this manner, five occurring in his own practice, two in that of Dr. Crawford; Chassaignac, Velpeau, and Nélaton, having each had one case. The oldest patient was thirteen, the youngest a new-born child, and in four there also existed chronic hydrocephalus.

Of the ten cases thus treated, five were cured, three died, and in two the results are uncertain, having occurred in the practice of MM. Nélaton and Velpeau. It is proper to state, however, that in one of the fatal cases hydrocephalus coexisted, and was the probable cause of this termination, as the spinal canal was found closed, and there was no irritation about the tumor to account for death. In another fatal case the spina bifida was cured, and the child died seven months afterwards from hydrocephalus. The result cannot, therefore, be attributed to the injections of iodine.

Dr. Brainard deems interference improper when the tumor is small, and the patient is somewhat advanced in years and in good health. Also in children affected with acute disease, especially of the brain and spinal cord.

The rules adopted by the author in making these injections are as follows:—

1. Make the puncture in the sound skin at the side of the tumor.

2. Inject the solution of iodine and iodide of potassium, commencing with one-fourth of a grain of the former to three-fourths of a grain of the latter, and retain it by slight pressure.

3. Evacuate no more of the liquid than the quantity of injection about to be thrown in.

4. If convulsions supervene, the fluid may be drawn out and its place filled with distilled water.

5. Lay the patient on the face, and if there be heat apply evaporating lotions to the tumor and to the head.

6. When the tumor becomes flaccid, apply collodion or other means of contracting the skin. This should be continued for some months after the swelling has disappeared.

7. After the effect of an injection is past, repeat it as many times as may be necessary, and gradually increase the strength according to the effect produced.

VIII.—*On Local Sweating of the Eyelids.* By PROFESSOR VON GRAFE. (Gräfe's Archiv, Band iv. p. 254.)

The author has observed this occurrence in four instances to an excessive extent, and in several others in a less degree. Patients thus affected appear, on superficial examination, to be the subjects of an ordinary conjunctivitis with excoriation of the lids; but the disproportion between the slight irritation of the conjunctiva and the great amount of secretion on the surface of the lid is at once obvious. The external surface of the lids, and especially of the upper one, is somewhat discolored and covered with a layer of fluid, the moisture being confined within the limit of the orbital ridge.

In Dr. Gräfe's first case, immediately after drying the lid, another thin layer of fluid was reproduced, so that even with the greatest care the skin could be kept dry only for a few minutes. The fluid, at first, was transparent, but gradually became turbid; and an examination with a lens showed that it issued from many minute, punctiform apertures in the skin, and finally collected in drops.

The conjunctivitis in these cases is only secondary, arising from the sweat flowing from the upper lids into the angles of the eyes. In two of the cases the affection was connected with general ephidrosis, and was increased by much exercise. It is an exceedingly obstinate disease, and is liable to be mistaken for conjunctivitis. Local applications of lead, nitrate of silver, and other articles, temporarily heal up the excoriations, but exert no influence on the secretion. In one case, smearing the lids with pitch was found to be beneficial. Cold baths, friction, and other means which induce activity of the skin, are important adjuvants in the treatment.

